# Fluorescence lifetime imaging microscopy reveals sodium pump dimers in live cells

**DOI:** 10.1016/j.jbc.2022.101865

**Published:** 2022-03-24

**Authors:** Jaroslava Seflova, Nima R. Habibi, John Q. Yap, Sean R. Cleary, Xuan Fang, Peter M. Kekenes-Huskey, L. Michel Espinoza-Fonseca, Julie B. Bossuyt, Seth L. Robia

**Affiliations:** 1 Department of Cell and Molecular Physiology, Loyola University Chicago, Maywood, Illinois, USA; 2 Department of Pharmacology, University of California Davis, Davis, California, USA; 3 Division of Cardiovascular Medicine, Department of Internal Medicine, Center for Arrhythmia Research, University of Michigan, Ann Arbor, Michigan, USA

**Keywords:** sodium pump, regulation, phospholemman, heart failure, FRET, dimerization, FXYD protein, protein-protein interactions, docking, molecular dynamics, AU, arbitrary units, CFP, cyan fluorescent protein, NKA, Na/K-ATPase, SERCA, sarco-endoplasmic reticulum Ca2+-ATPase, TCSPC, time correlated single photon counting, YFP, yellow fluorescent protein

## Abstract

The sodium-potassium ATPase (Na/K-ATPase, NKA) establishes ion gradients that facilitate many physiological functions including action potentials and secondary transport processes. NKA comprises a catalytic subunit (alpha) that interacts closely with an essential subunit (beta) and regulatory transmembrane micropeptides called FXYD proteins. In the heart, a key modulatory partner is the FXYD protein phospholemman (PLM, FXYD1), but the stoichiometry of the alpha–beta–PLM regulatory complex is unknown. Here, we used fluorescence lifetime imaging and spectroscopy to investigate the structure, stoichiometry, and affinity of the NKA-regulatory complex. We observed a concentration-dependent binding of the subunits of NKA–PLM regulatory complex, with avid association of the alpha subunit with the essential beta subunit as well as lower affinity alpha–alpha and alpha–PLM interactions. These data provide the first evidence that, in intact live cells, the regulatory complex is composed of two alpha subunits associated with two beta subunits, decorated with two PLM regulatory subunits. Docking and molecular dynamics (MD) simulations generated a structural model of the complex that is consistent with our experimental observations. We propose that alpha–alpha subunit interactions support conformational coupling of the catalytic subunits, which may enhance NKA turnover rate. These observations provide insight into the pathophysiology of heart failure, wherein low NKA expression may be insufficient to support formation of the complete regulatory complex with the stoichiometry (alpha-beta-PLM)_2_.

The sodium pump (NKA, Na^+^/K^+^-ATPase) is an essential membrane-bound transport ATPase establishing vital Na^+^ and K^+^ gradients across the plasma membrane. During its reaction cycle, NKA uses energy from ATP hydrolysis to translocate three Na^+^ ions from the cytoplasm into the extracellular space and two K^+^ from outside the cell into the cytoplasm. NKA comprises a complex of the α catalytic subunit that binds substrate ions and ATP and a β subunit that is critical for correct localization of the α subunit in the plasma membrane. Na^+^ transport is finely regulated by tissue-specific expression of four different α subunit isoforms with three different β subunit isoforms. Additional homeostatic control is provided by interactions of the essential αβ complex with seven diverse regulatory partners from the FXYD family of micropeptides (1, 2). FXYD proteins contain a single transmembrane helix and a highly conserved extracellular extension containing a FXYD motif, which gives this family of NKA regulators its name. In the heart, NKA is regulated by an inhibitory interaction with FXYD1, also known as phospholemman (PLM) (3). This inhibition results in a comparatively higher intracellular Na^+^ concentration, limiting Ca^2+^ extrusion by the Na^+^/Ca^2+^ exchanger and thereby increasing intracellular Ca^2+^. Increased Ca^2+^ handling supports stronger cardiac contractions. This physiological connection between cardiac Na^+^ transport and Ca^2+^ handling provides a mechanism of action for digitalis, an NKA-inhibiting drug used for hundreds of years to increase cardiac contractility in patients with heart failure (4, 5).

X-ray crystallography revealed a NKA-regulatory complex with a concise stoichiometry of one FXYD protein and one β subunit bound to a single α subunit. However, biochemical studies suggest that the subunits may assemble in a larger functional complex. Initial work by Stein *et al.* (6), Ottolenghi and Jensen (7) suggested NKA dimerization based on cooperative ATP hydrolysis, which may indicate a catalytic dimer of functionally coupled α subunits (8). Electrophoretic analysis (9, 10) was also consistent with a complex of two α subunits. NKA oligomerization has been invoked to explain other experimental anomalies such as NKA ouabain-digoxin antagonism (11, 12, 13). That is, similar pharmacological inhibitors may show opposing effects that could be explained by functional interactions of protomers of a multimeric transporter complex. Functional dimerization has also been observed for other members of P-type ATPase family (14, 15, 16, 17, 18, 19, 20, 21), including the closely related ion pump, sarco-endoplasmic reticulum Ca^2+^-ATPase (SERCA) (22, 23, 24). Kinetic studies of that transporter revealed that conformational coupling within the dimer complex enhances overall enzyme turnover by linking an energetically unfavorable step of one SERCA protomer to an energetically favorable step of the other protomer (22, 25). Whether this biochemical mechanism is common to other oligomeric transporters is unknown.

In this study, we probed the stoichiometry of human NKA-PLM regulatory complex using fluorescence spectroscopy and computational simulations to evaluate alternative arrangements of subunits. The results provide insight into the quaternary architecture of the complex, with implications for a structural mechanism of conformational coupling of the catalytic subunits.

## Results

### The quaternary structure of NKA-PLM regulatory complex expressed in HEK cells

**Figure 1:**
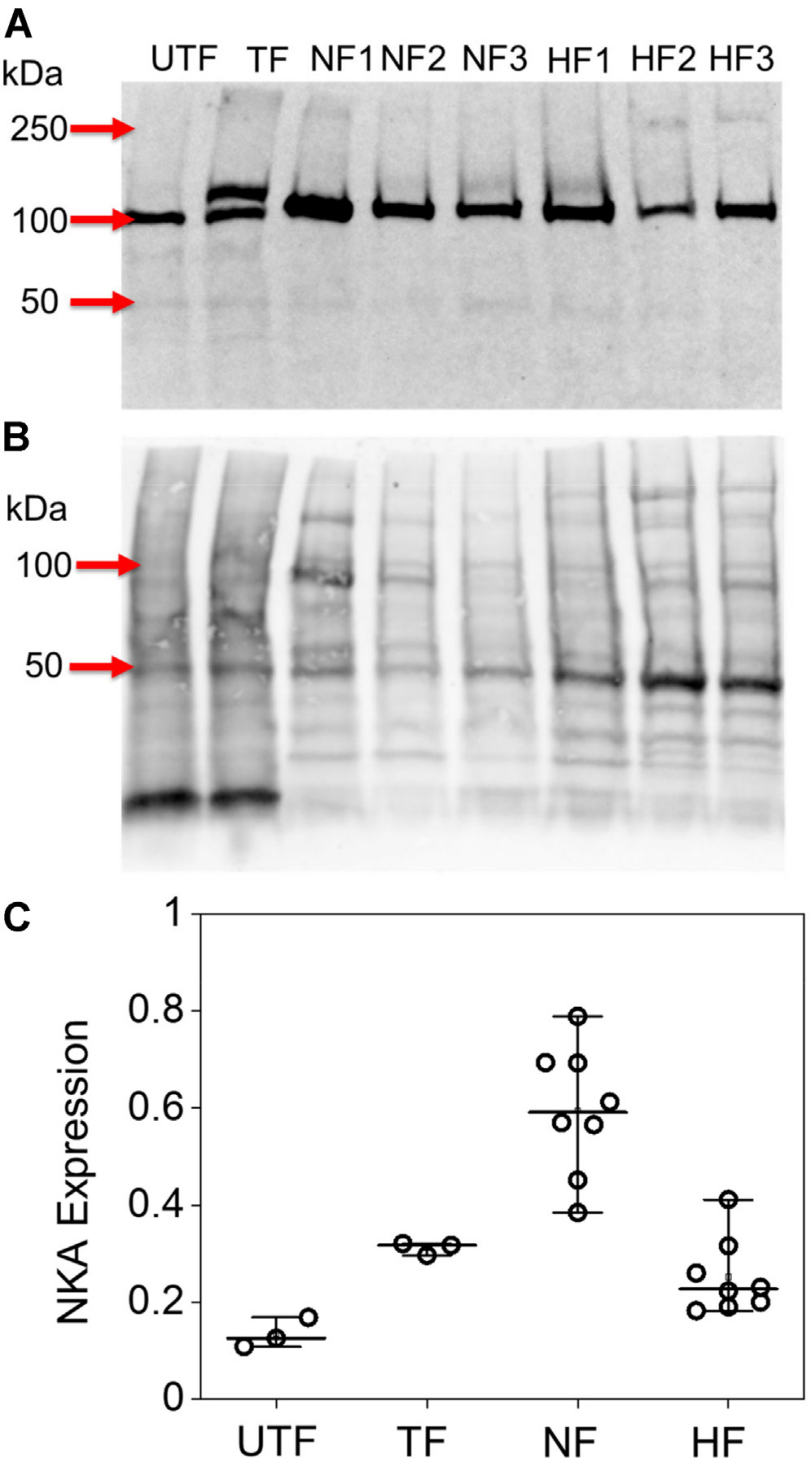
**Western blot analysis of sodium pump expression in heterologous cells and myocardium.***A*, untransfected HEK cell microsomal fractions (UTF) showed endogenous NKA with an apparent MW of 100 kDa. Microsomes from cells transfected with human mCer-α_1_ NKA (TF) showed bands representing endogenous NKA and exogenous fluorescent NKA, which showed a decreased mobility due to the 30 kDa tag. NKA was highly expressed in microsomal fractions isolated from human myocardium. We observed decreased expression of NKA in failing human heart (HF1-3) compared to nonfailing hearts (NF1-3). *B*, total protein staining (Revert). *C*, quantification of NKA expression in HEK cells and human nonfailing and failing hearts, normalized to total protein. TF and UTF data represent three independent transfections. NF and HF data represent tissue samples from eight nonfailing donor hearts and eight explanted hearts with dilated cardiomyopathy. NKA, Na/K-ATPase.

To investigate the interactions of catalytic and regulatory subunits in the NKA complex, we expressed fluorescently labeled proteins in HEK AAV-293 cells using transient transfection. To compare the protein expression levels between heterologous expression and expression of endogenous NKA in human myocardium, we performed Western blotting of microsomal fractions (Fig. 1, *A* and *B*) and quantified NKA content with normalization to total protein (Fig. 1*C*). Endogenous NKA α_1_ with an electrophoretic mobility of ∼100 kDa was detected in the membrane fractions obtained from untransfected (Fig. 1, *A* and *C*) HEK AAV-293 cells. Transient transfection of fluorescently labeled pump resulted in an additional higher molecular weight band of intensity similar to endogenous protein (Fig. 1, *A* and *C*). Thus, transfection approximately doubled the concentration of NKA in the cell membranes. By comparison, microsomes obtained from nonfailing human myocardium showed approximately 2-fold higher expression of NKA. In agreement with previous studies, we found NKA expression in the failing myocardium was reduced (26, 27), and NKA expression was similar to that of transfected HEK cells (Fig. 1, *A* and *C*). We conclude that heterologous expression of NKA yields physiological protein levels that are comparable to the diminished NKA concentration in the failing human heart. Some samples manifested a faint higher molecular weight band with mobility ∼250 kDa. This band may represent an oligomeric α species that persisted through solubilization and electrophoresis. An uncropped image of the blot is shown in Fig. S1.

**Figure 2:**
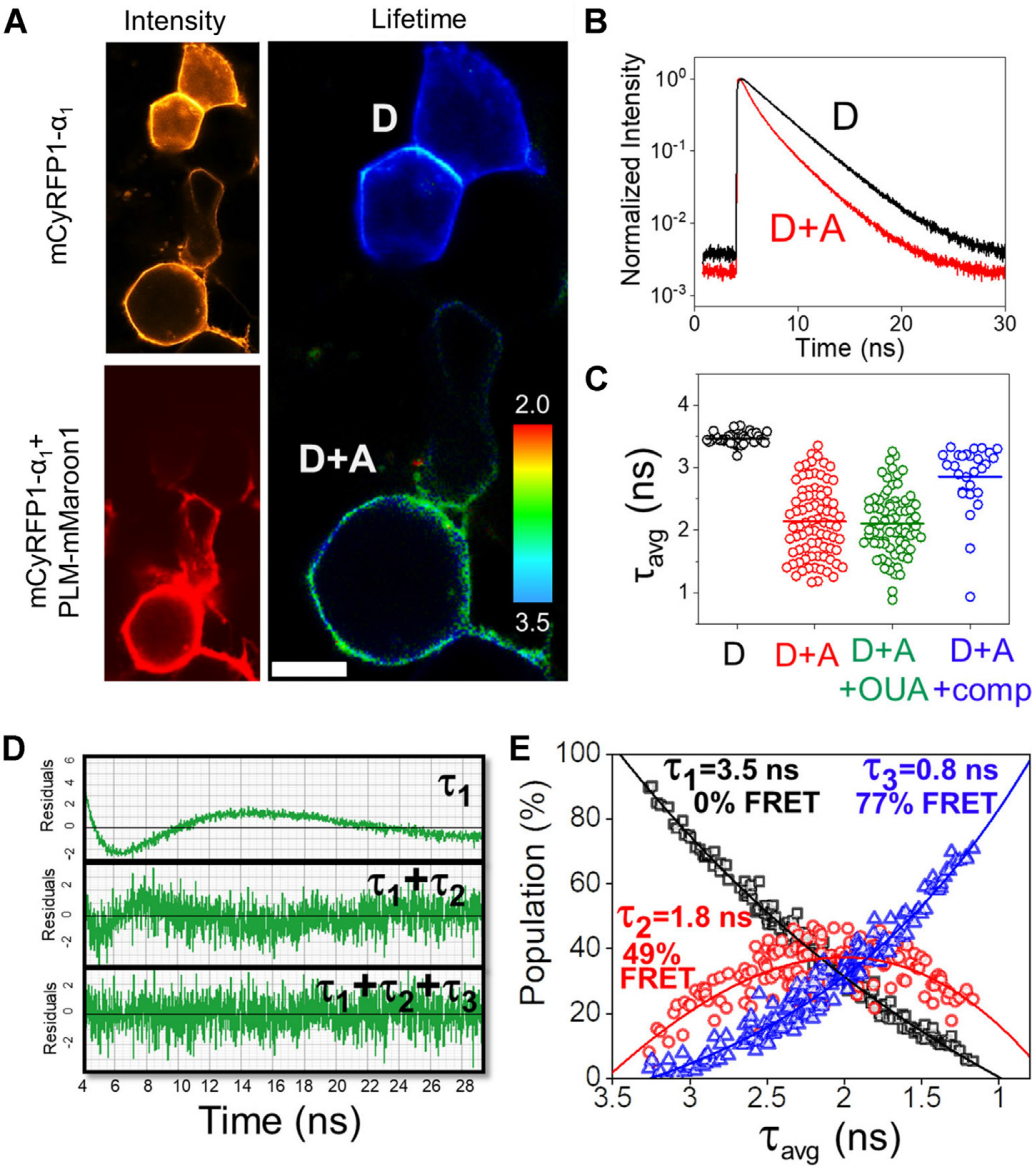
**Quantification of the α**_**1**_**-PLM interaction using TCSPC.***A*, fluorescence intensity and fluorescence lifetime data acquired by FLIM. Cells expressing only mCyRFP1-α_1_ (donor, D) were characterized by a long lifetime. Cells coexpressing PLM-mMaroon1 (donor + acceptor, D + A) showed shorter lifetimes. The scale bar represents 10 μm. *B*, fluorescence decay curves for donor alone (D) and donor + acceptor (D + A). *C*, comparison of amplitude-weighted average fluorescence lifetimes obtained by multi-exponential fitting of decay data. The donor lifetime (*black*, D) was shortened by FRET with the acceptor (*red*, D + A). The addition of 10 μM ouabain (*green*, OUA) did not affect detected FRET. FRET was decreased by competition with unlabeled PLM (*blue*, D + A + comp). *D*, a plot of residuals of exponential decay fitting showed the fit was improved by the addition of second and third components, suggesting three fluorescent species with distinct lifetimes. *E*, the relative population of the three decay components depended on the overall average lifetime of the decay. Those components are attributed three species with FRET efficiencies of 0% (*black*), 49% (*red*), and 77% (*blue*). FLIM, fluorescence lifetime imaging microscopy; TCSPC, time correlated single photon counting.

We performed fluorescence lifetime microscopy to quantify FRET between NKA α subunit and PLM. We engineered fusion constructs with the FRET donor mCyRFP1 (22, 28, 29) attached to the N-terminus of NKA α subunit and attached mMaroon1 (29) (FRET acceptor) to the C-terminus of PLM (on the cytoplasmic side), expressing these fusion constructs at a 1:5 ratio, together with unlabeled β_1_ subunit. Figure 2*A* shows that cells expressing both the donor and acceptor (D + A) were characterized by a significantly shortened fluorescence lifetime (τ ∼ 2 ns) compared to cells expressing only the donor fluorophore (D), which had a fluorescence lifetime of 3.5 ns. The data are consistent with the shortening of τ by FRET. For a more detailed analysis of the fluorescence decay, we performed time-correlated single photon counting (TCSPC) point measurements, parking the excitation beam at a single spot on the plasma membrane to collect ∼10^6^ photons for each record, as previously described (22, 23). Cells expressing mCyRFP1 (nonfusion) showed a single exponential decay with a characteristic decay time (τ) of 3.51 ns (Fig. S2), and mCyRFP1-α_1_ was also well described by a single exponential decay (Fig. 2*B*, black trace, “D”), with a τ of 3.46 ns (Fig. 2*C*, black points, “D”) (see Experimental procedures). Coexpression of PLM-mMaroon with mCyRFP1-α_1_ decreased the mCyRFP1-α_1_ lifetime, yielding a more complex decay (Fig. 2*B*, red, “D + A”) with average lifetime (τ_avg_) values ranging from 1 to 3 ns (Fig. 2*C*, red, “D + A”). α-PLM FRET was not affected by 10 μM ouabain (Fig. 2*C*, green, “D + A + OUA”). To determine whether α-PLM FRET was due to a specific interaction of donor-labeled α_1_ with acceptor-labeled α_1_, we coexpressed an excess of unlabeled α_1_, resulting in a significant increase in τ_avg_ (Fig. 2*C*, blue “D + A + comp”). The data suggest that the observed mCyRFP1-mMaroon FRET is due to a specific physical interaction of the α_1_ and PLM, rather than nonspecific FRET between fluorophores in the crowded plasma membrane. We observed similar results for α_2_ and α_3_ (Fig. S3, *A*–*C*).

The more complex decay of the D + A expressing cells was not well described by a single exponential function, as is evident from a plot of the fit residuals (Fig. 2*D*, τ_1_). Addition of a second and a third component improved the quality of the fit (Fig. 2*D*) but adding a 4th decay component did not appreciably improve the residuals plot or reduced chi-squared value. Thus, the data support the existence of three species with well-resolved fluorescence lifetimes: τ_1_ = 3.46 ns, which we attribute to donors not interacting with acceptors; τ_2_ = 1.8 ns, which suggests a population of donors undergoing FRET with 49% efficiency; τ_3_ = 0.8 ns, which corresponds to a high FRET population (77% FRET). The relative populations of these species varied from cell to cell, and we noted increased contribution of high FRET species in brightly fluorescent cells expressing a high concentration of protein. Indeed, the overall fluorescence lifetime (τ_avg_) is inversely related to cell fluorescence, suggesting increasing FRET at higher protein expression levels. The concentration dependence of FRET is analyzed in more detail below.

Figure 2*E* shows the results of exponential fitting of decays obtained from a survey of ∼200 cells, expressing a wide range of protein concentrations. The amplitude of each component lifetime (τ_1_, τ_2_, and τ_3_) varied systematically with the value of the overall decay lifetime (τ_avg_). Cells yielding decays with longer average lifetimes (τ_avg_) indicating low FRET had strong contributions from the donor-only component (τ_1_) and low populations of the FRET species (τ_2_, τ_3_). These cells tended to be lower-expressing, dimly fluorescent cells in the population. Data from these cells falls on the left side of Figure 2*E*. Conversely, brightly fluorescent cells tended to have short decays (low τ_avg_), falling on the right side of Figure 2*E*. These cells showed strong contributions from the high FRET species (Fig. 2*E*, blue triangles) and little contribution from the donor-alone species (black squares). Interestingly, the medium FRET species (red circles) showed a biphasic dependence on τ_avg_, with low contribution to very long and very short decays and a maximal population in decays with τ_avg_ near 2 ns. We observed the same FRET-dependent populations of species for α_1_, α_2_, and α_3_ (Fig. S3, *D*–*F*), so data from all α isoforms are pooled and presented together in Figure 2*E*.

**Figure 3:**
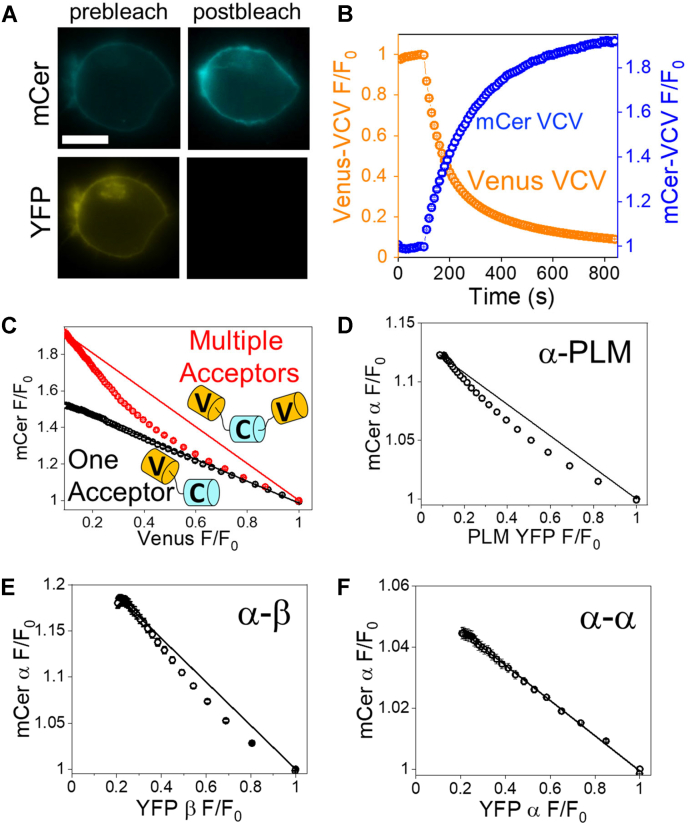
**Progressive acceptor photobleaching provides insight into regulatory complex stoichiometry.***A*, images of cells expressing NKA and PLM labeled with mCerulean (mCer) and YFP, respectively, before and after acceptor selective photobleaching. The scale bar represents the size of 10 μm. *B*, in a control experiment, donor (mCer) fluorescence intensity increased as acceptor (Venus) was progressively photobleached. *C*, replotting data (as in *B*) reveals the relationship between donor and acceptor fluorescence intensity. The relationship is linear when one acceptor is present, for example, mCer separated from Venus by five amino acids (C5V, *black*) but is curved when there are multiple acceptors participating in FRET (VCV, *red*). *D*, the regulatory complex contains multiple acceptor-labeled PLM in complex with donor-labeled α (n = 56). *E*, the complex contains multiple acceptor-labeled β (n = 42). *F*, the α-α complex produced a linear donor/acceptor relationship, suggesting a single acceptor-labeled α in complex with donor-labeled α (n = 150). NKA, Na/K-ATPase; YFP, yellow fluorescent protein.

To understand the nature of the three distinct FRET species, we sought to systematically investigate the relative population of each component as a function of overall average fluorescence lifetime (τ_avg_), which was inversely related to acceptor expression. We considered that the high FRET species may represent an alternative conformation in which the donor and acceptor were in closer proximity, however, the concentration-dependent appearance of this component suggests that it represents a higher-order complex of NKA and PLM, containing more than one acceptor-labeled PLM. To evaluate this possibility, we performed progressive acceptor photobleaching of cells expressing mCer-NKA and PLM-yellow fluorescent protein (YFP). This approach is illustrated in Figure 3*A*: destruction of the YFP (acceptor) abolishes FRET, dequenching the mCer (donor). Stepwise photobleaching over time while monitoring the donor and acceptor brightness reveals the time-dependent changes in these signals (Fig. 3*B*) and plotting these signals against each other reveals the relationship between donor and acceptor fluorescence intensity (Fig. 3*C*). This donor/acceptor relationship indicates whether there is a single acceptor in the FRET complex or multiple acceptors. The standard FRET fusion construct (30) C5V shows a linear plot of donor *versus* acceptor brightness (Fig. 3*C*, black), while a control fusion construct with two acceptors (VCV) shows pronounced curvature (Fig. 3*C*, red) (23, 31, 32, 33, 34). This curvature occurs because FRET persists after bleaching the first acceptor in the complex, thus dequenching of the donor is delayed until later in the progressive photobleaching time course. Subjecting the mCer-NKA+PLM-YFP expressing cells to progressive acceptor photobleaching yielded a donor/acceptor relationship with significant curvature (Fig. 3*D*), indicating that the donor-labeled NKA is in a complex with more than one acceptor-labeled PLM. The Discussion section evaluates this noteworthy observation in the context of previous similar studies.

To develop a fuller picture of the NKA-regulatory complex subunit stoichiometry, we engineered an acceptor-labeled β subunit and coexpressed it with donor-labeled α subunit. Progressive acceptor photobleaching yielded a nonlinear D/A plot (Fig. 3*E*), suggesting the NKA complex contains multiple β subunits. Finally, we tested whether the complex included multiple α subunits by quantifying FRET from mCer-α_1_ to YFP-α_1_. We performed progressive acceptor photobleaching and observed a linear relationship (Fig. 3*F*) suggestive of a single acceptor-labeled α protomer in complex with the donor-labeled α protomer. Collectively, these data suggest that the NKA complex includes an α-α homodimer decorated with multiple β subunits and multiple FXYD proteins. The Discussion section describes how this insight into α:β:FXYD stoichiometry may be used to discriminate between alternative models of the regulatory complex quaternary architecture. In contrast to other reports that indicated deoligomerization of NKA in the presence of the NKA inhibitor ouabain (13), we did not see a decrease in FRET in the presence of 10 μM ouabain, whether FRET was measured by acceptor sensitization FRET or TCSPC (Fig. S3, *A*–*C*). Coexpression of unlabeled PLM did not increase or decrease α-α FRET measured by TCSPC (Fig. S4, *A* and *B*).

**Figure 4:**
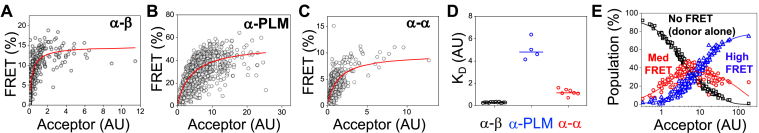
**Quantification of binding affinity with acceptor sensitization FRET.***A*, the α-β FRET binding curve revealed a very high affinity interaction. *B*, the apparent affinity of α for PLM was lower. *C*, α-α binding affinity was intermediate. *D*, summary of measured dissociation constants for α-β (*black*), α-PLM (*blue*), and α-α (*red*) interactions. *E*, indexing lifetime data from Figure 1*E* with acceptor sensitization FRET measurements in Figure 3*B* reveals concentration-dependent assembly of distinct FRET species.

The apparent concentration-dependent appearance of different FRET species motivated a comparison of the relative affinities of the interaction of the NKA α subunit with other components of the complex. We quantified FRET from a heterogeneous population of cells using the acceptor sensitization method and also quantified acceptor fluorescence emission as an index of protein concentration in the cell, as previously described (22, 33, 34, 35, 36, 37, 38). A plot of observed FRET *versus* the cell acceptor fluorescence emission revealed the concentration dependence of FRET, which was well described by a hyperbolic function of the form $FRET_{observed}=\frac{FRET_{max}\left[protein\right]}{K_{D}+\left[protein\right]}$, where FRET_max_ is the maximal FRET obtained at the highest concentrations and K_D_ is the apparent dissociation constant, the protein concentration that yielded half-maximal FRET (34). The concentration-dependence of FRET from donor-labeled α subunit to acceptor-labeled β suggested a very avid interaction (Fig. 4*A*), with an apparent dissociation constant that was too low to quantify reliably (K_D_ < 0.5 arbitrary units (AU)). We conclude that the α–β interaction is constitutive under these experimental conditions. The FRET-based binding curve for α and PLM revealed a lower affinity interaction (Fig. 4*B*), with a higher apparent K_D_ (5.0 ± 0.8 AU). Importantly, the PLM-binding affinity is underestimated in this assay because PLM forms tetramers that do not interact with the α subunit (39). Thus, the true concentration of monomeric PLM that is available to bind α is somewhat less than what would be estimated from total YFP-PLM fluorescence intensity. We also observed FRET from donor-labeled α to acceptor-labeled α (Fig. 4*C*), with an apparent K_D_ of 1.2 ± 0.3 AU, consistent with the acceptor photobleaching experiments that suggested the NKA complex contains an α-α dimer (Fig. 3*F*). The relative dissociation constants of the α subunit interactions with other subunits of the NKA complex are summarized in Figure 4*D*.

The observed α-PLM acceptor sensitization FRET binding curve (Fig. 4*B*) revealed the relationship between protein expression and FRET efficiency. This relationship was used to index τ_avg_ values (Fig. 2*E*, x-axis values) to protein concentration after converting τ_avg_ to observed FRET, according to the relationship $FRET_{observed}=1-\frac{{\text{τ}}_{avg}}{{\text{τ}}_{D}}$. Replotting of the data of Figure 2*E* as a function of relative protein concentration revealed that the NKA–PLM interaction is low in the lowest expressing cells, increasing as the protein concentration grows (Fig. 4*E*). The non-FRET species (Fig. 4*E*, black squares, τ_1_) that predominates at low protein expression levels consists of donor-labeled α that is not bound to acceptor-labeled PLM. However, even at these low protein concentrations, the α–β interaction occurs since this interaction has the highest relative affinity (Fig. 4*A*) and may be considered constitutive. At higher concentrations, the higher order complexes begin to form. In particular, the highest FRET species (Fig. 4*E*, blue triangles, τ_3_ = 0.8 ns, FRET = 77%) is the dominant complex at the highest concentrations. Curvature analysis (Fig. 3) suggests this species includes multiple β subunits and multiple PLM in the complex, and it occurs at protein concentrations where α–α interactions are observed (Fig. 4*C*). The possible stoichiometry of subunits and concentration-dependent assembly pathway for the regulatory complex are discussed below.

### Hetero-dimerization of different α subunit isoforms

**Figure 5:**
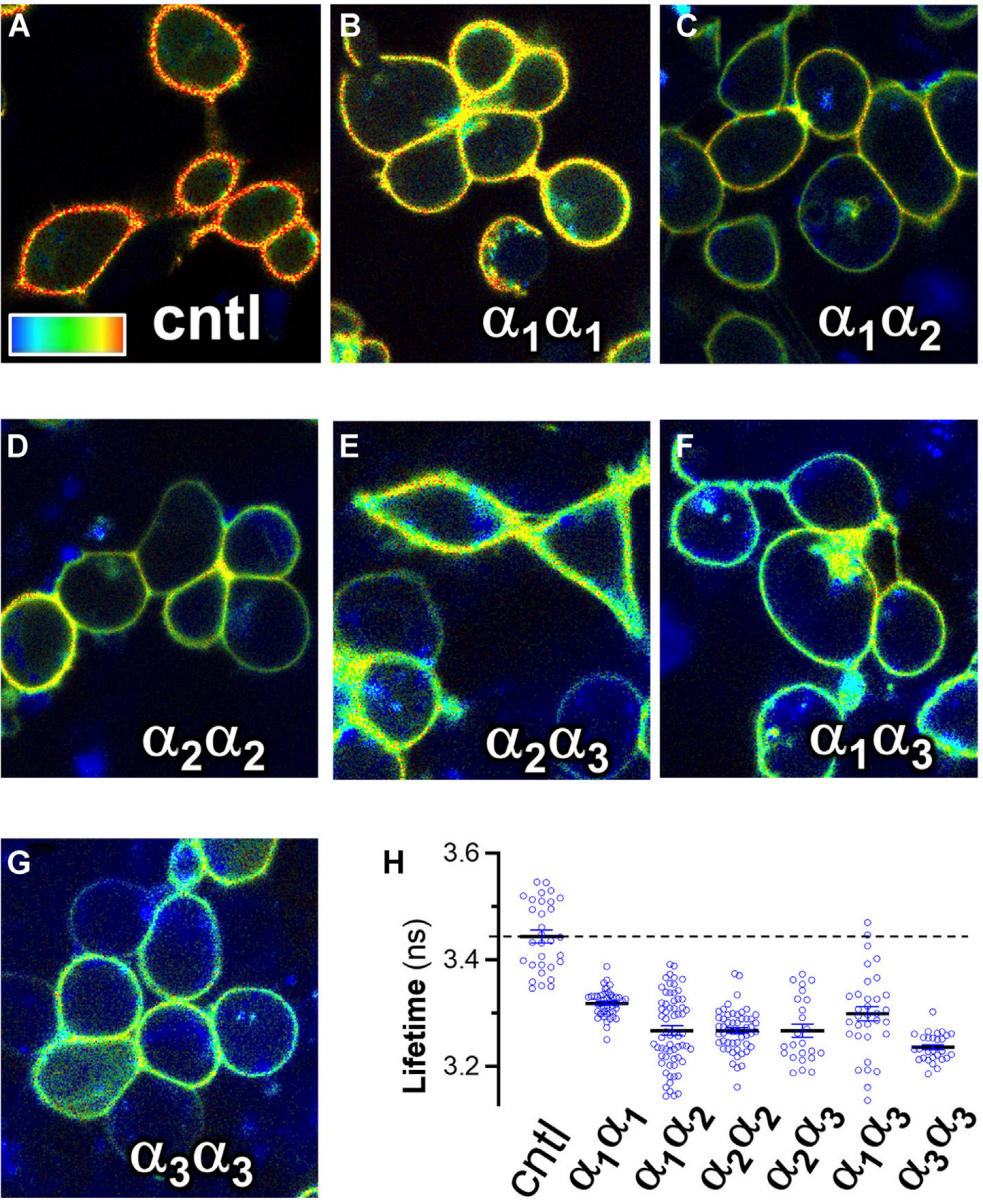
**FLIM analysis of α-α FRET.***A*, control (donor alone). *B*–*G*, combinations of α subunit isoforms. *H*, quantification of FLIM images revealed all combinations of isoforms support FRET, measured as decreased lifetime compared to donor alone control (*dotted line*). The data suggest α-α interactions are a general mechanism. The color scale represents 2.2 ns (*blue*) to 3.4 ns (*red*) and 25 μm in size. FLIM, fluorescence lifetime imaging microscopy.

In this study, we primarily studied the housekeeping isoform α_1_ in evaluating NKA–PLM complex stoichiometry. To determine whether all isoforms support α–α interactions or if the interaction is specific to the α_1_ isoform, we fused mCyRFP1 and mMaroon1 to the three main human α isoforms and acquired fluorescence lifetime imaging microscopy data from different combinations of these α subunits. We observed the expected plasma membrane localization for the labeled proteins (Fig. 5, *A*–*G*), and we noted a decreased donor fluorescence lifetime for every combination of isoforms (Fig. 5*H*), consistent with FRET between donor- and acceptor-labeled α subunits (Fig. S4, *A* and *B*, red). Thus, α–α interactions appear to be a general feature of all three α isoforms.

### Molecular docking of NKA α subunit

To interpret FRET distance measurements in the light of available high resolution structural information, we performed protein-protein docking simulations to hypothesize atomistic structures of the quaternary complex formed by NKA α-β-PLM. Docking simulations were performed using crystal structures of NKA that captured the E1 state of the pump (PDB: 3WGU (40)). The complexes were obtained with Rosetta MPDock (41, 42), generating 1000 configurations that were ranked based on the Rosetta interaction energy units. Inspection of the top ranked complexes yielded three configurations deemed to be plausible based on (1) correct topological orientation, (2) a substantial interaction surface, and (3) compatibility with estimates of FRET distances between α–α, α–PLM, and α−β labeling sites. These complexes were minimized and subjected to molecular dynamics (MD) simulations for between 0.8 μs and 1 μs.

**Figure 6:**
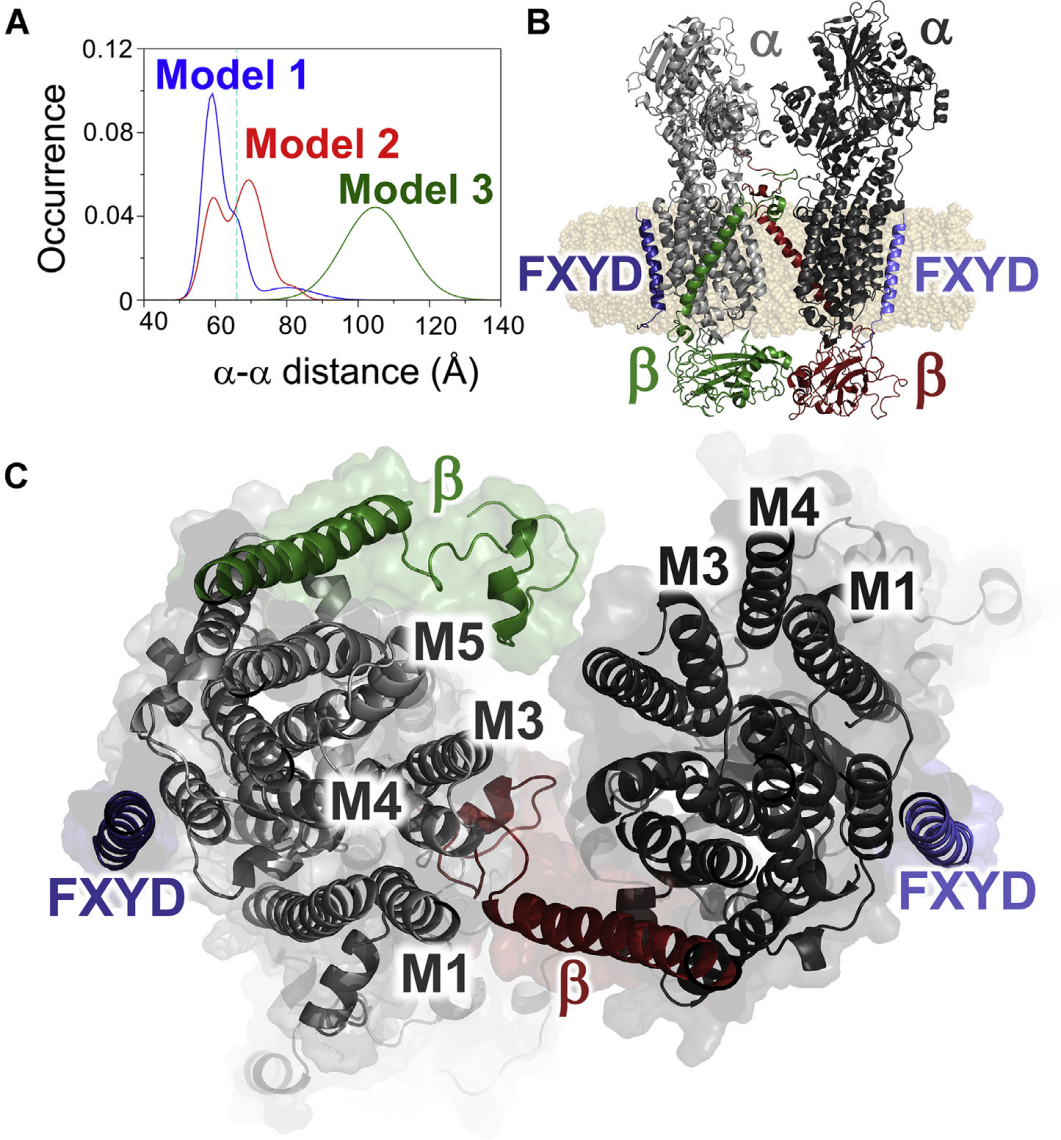
**Comparing possible α-α dimer arrangements.***A*, the distribution of distances between α subunit N-termini sampled during molecular dynamics (MD) simulations. *B*, structural Model 1, showing docked E1P conformations of the α subunits. α subunits are shown in *light gray* and *dark gray*, β subunits are *green* and *red*; and the FXYD proteins are shown in *blue* and *lavender*. *C*, intramolecular contacts in Structural Model 1, viewed from the extracellular side.

Analysis of the MD trajectories showed that the three dimers remained structurally stable and do not dissociate on this time scale (Fig. S5). We compared the distance between the N-termini of the docked α subunits with that estimated from FRET experiments to determine which model best represents the structure of the complex. We found that Structural Model 1 and 2 yield α-α distance distributions (Fig. 6*A*, blue, red) that were comparable to the distance estimated from quantification of FRET, 65 Å (Fig. 6*A*, dotted line). The distance distribution for Structural Model 1 reflects a stable, relatively compact architecture, as illustrated by a single peak centered at 60 Å (Fig. 6*A*). Structural Model 2 sampled a range of distances that distributed between two peaks at 60 Å and 70 Å. Compared to these models, Structural Model 3 exhibited substantially longer distances between α subunit N-termini (*R* = 105 Å, Fig. 6*A*, green). This distance distribution is not well matched to apparent fluorescent probe separation distance estimated by FRET. However, the fluorescent protein tags are large and connected by a flexible linker to the α subunit N-terminus, so we cannot rule out Structural Model 3. A comparison of alternative Structural Model 1, 2, and 3 is provided in Fig. S6, *A*–*F*.

The arrangement of subunits in Structural Model 1 is shown in Figure 6*B*, with α subunit protomers shown in light and dark gray, the β subunits shown in green and red, and the FXYD proteins shown in blue and lavender. The complex is oriented with the α subunit cytoplasmic domains at the top of the figure. We noted intermolecular contacts between extracellular domains of both β subunits and between the A- and P-domains of the α subunits. We also observed contacts between the N-terminus of the β subunit and and transmembrane helix 3 (M3) of each adjacent α subunits. These contacts examined in more detail in Figure 6*C*, which shows a section through the center of the Structural Model 1, as viewed from the extracellular side.

## Discussion

### The architecture of the NKA-regulatory complex

**Figure 7:**
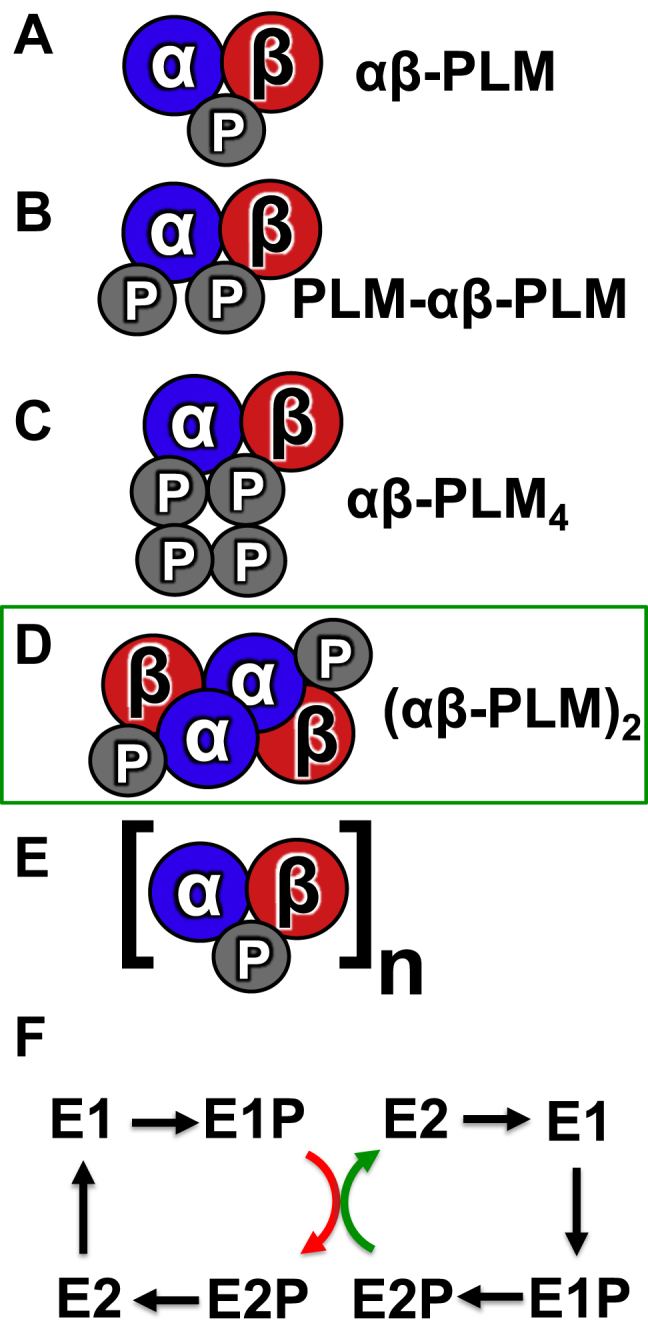
**The stoichiometry, assembly, and function of the NKA-regulatory complex.***A*–*E*, alternative models of NKA-regulatory complex stoichiometry. The present data are consistent with Stoichiometry Model D. *F*, a model of conformational coupling in a dimer of α subunits. An energetically favorable catalytic step of one protomer (*green arrow*) assists a rate limiting step (*red arrow*) of the other partner, enhancing overall turnover. NKA, Na/K-ATPase.

Previous studies employing diverse experimental approaches have provided evidence of α subunit oligomerization in the NKA-regulatory complex (11, 12, 13, 23, 43, 44, 45). Here, we explored the binding affinity between NKA subunits and investigated the stoichiometry of the NKA-regulatory complex. We considered several possible alternative stoichiometries for the regulatory complex, depicted in the schematic diagram of Figure 7, *A*–*E*. Possible arrangements include the following: Stoichiometry Model A, a simple complex containing a single protomer of each type (αβ-PLM); B, αβ binds two PLM proteins at two distinct sites (high and low affinity) (PLM-αβ-PLM); C, αβ binds a PLM tetramer (33, 39, 46, 47) (αβ-PLM_4_); D, two αβ complexes bind to each other, along with regulatory PLM partners (αβ-PLM)_2_; and E, which is higher order oligomerization/aggregation (13). The TCSPC measurements (Fig. 2*E*) and progressive acceptor photobleaching experiments (Fig. 3*D*) rule out the simplest model, Model A (Fig. 7*A* “αβ-PLM”). Next, progressive photobleaching quantification of α-β FRET revealed a curved D/A plot that indicates multiple β subunits in the complex (Fig. 3*E*). This observation rules out Stoichiometry Models A, B, and C, which do not have multiple β subunits. Finally, we measured α-α FRET (Fig. 3*F*) with progressive photobleaching and observed a linear plot, which shows the regulatory complex contains two α subunits. This observation therefore rules out Stoichiometry Models A, B, C, and E. We propose that Stoichiometry Model D, characterized by the complex of two α catalytic subunits plus regulatory partners (αβ-PLM)_2_, best describes the architecture of the NKA-regulatory complex.

Complementary atomistic simulations produced several structural models of the dimer that agree with Stoichiometry Model D. The structural models all showed a high degree of symmetry (Fig. S6), which is a common characteristic of stable, multivalent, allosteric complexes (48). The simulations suggest an interaction between the N-termini of β and helix M3 of the α subunit, which is noteworthy because helix M3 is highly conserved across species. Mutations in this region induce changes in the functional E1-E2 transitions, alter Na^+^ binding to the transport sites, and affect the allosteric interactions that control NKA dephosphorylation (49). The functional significance of this region is also emphasized by the association between mutations in the helix M3 and neurological diseases such as familial hemiplegic migraine and renal hypomagnesemia (50, 51, 52). We speculate that changes in α subunit functional dimerization may contribute to the deleterious effect of pathological M3 mutations.

### The functional significance of NKA-regulatory complex stoichiometry

The possibility of a dimeric NKA was first raised in 1973 (6, 53), but the physiological function of the α–α interaction is still unclear. Apparent inter-protomer allosteric communication between ATP binding sites suggests a functional interaction between α subunits. Functional coupling may support synchronized cycling of pumps (12, 54) for enhanced transport kinetics. Alternatively, the mechanism of functional coupling may follow the pattern observed for another dimeric transporter, the SERCA Ca^2+^ pump. We (22, 23) and others (24) have observed SERCA–SERCA interactions; kinetic studies suggested that SERCA dimerization enhances Ca^2+^ transport through antisynchronous cycling of the protomers (55). In this “conformational coupling” model, dimerization serves to speed up rate-limiting steps in the catalytic cycle of one protomer by coupling them to thermodynamically favorable steps in the other protomer (Fig. 7*F*). We hypothesize that NKA, like SERCA, may benefit from an increased catalytic efficiency and faster turnover rate due to conformational coupling of α subunit protomers.

### Concentration-dependent assembly of the NKA-regulatory complex

The present progressive photobleaching experiments suggested that the regulatory complex contains more than one PLM, as shown in the pronounced curvature in the donor *versus* acceptor plot (Fig. 3*D*). This result was unexpected and contrasted with our previous progressive acceptor photobleaching measurements of canine or rat NKA α_1_ in complex with mouse PLM, in which we observed only modest curvature (38, 56). This apparent discrepancy may be due to a lower level of protein expression that was achieved in that previous study using a cyan fluorescent protein (CFP)-NKA stable cell line. The concentration-dependent assembly of lower and higher FRET NKA complexes (Fig. 4*E*) suggests that at lower protein expression, NKA forms a simpler complex containing a single acceptor, but at higher concentrations, a larger complex containing multiple acceptor-labeled PLM subunits prevails. This larger complex with multiple acceptors gives rise to the curved D/A relationship seen in the present study (Fig. 3*D*) and accounts for the observed high FRET species seen in TCSPC experiments (Fig. 4*E*). Similarly, we have previously observed stepwise assembly of oligomers of membrane micropeptides (34). In that study, we found dimers formed first, yielding a linear D/A plot at low protein concentration. Then, as expression increased, the D/A curvature increased suggesting higher order oligomerization.

In considering the biological significance of the observed concentration-dependent assembly of subunits, we note possible relevance for the pathophysiology of heart failure. In transfected HEK cells, we see a range of NKA and PLM expression levels. Cells in the middle of the distribution express protein at a level that supports ∼20% of the NKA population in the high FRET species (Fig. 4*E*). Comparing microsomal membrane preparations from HEK cells with membranes of cardiac myocytes suggests that NKA expression in the heart is more than 2-fold higher (Fig. 1). Consequently, we estimate that >60% of NKA in the heart is fully assembled in the high order complex with stoichiometry (αβ-PLM)_2_. However, under pathological conditions, such as in heart failure, NKA α subunit expression and PLM expression are significantly decreased (27). Indeed, we observed that membrane microsomes isolated from failing human myocardium expresses NKA α at low levels that are similar to the transfected HEK cell model system used here (Fig. 1). Under these pathological conditions, the low protein concentration may not be sufficient to support the assembly of functional regulatory complex with correct stoichiometry, (αβ-PLM)_2_. The K_D_ of the α–β interaction is 0.3 AU, so low that this avid interaction probably persists even at reduced α concentrations. However, the K_D_s of the α–PLM and α–α interactions were 5.0 and 1.2 AU, respectively. These values are similar to the average α expression level in HEK cells (1.0 AU) (Fig. 4), which is similar to α expression in the failing heart (Fig. 1). Thus, the α-PLM and α-α binding equilibria are more likely to be sensitive to changing α expression during heart failure.

### Summary

Collectively, our results are consistent with a sodium/potassium transporter regulatory complex that is composed of two α subunits associated with two β subunits and two PLM regulatory subunits. Docking and MD simulations illustrated several possible quaternary arrangements of these subunits, but confirmation of the true architecture of the (αβ-PLM)_2_ regulatory complex will require high resolution structure determination under conditions that preserve the higher-order oligomeric form of NKA. We propose that α–α interactions support conformational coupling of the catalytic subunits, enhancing NKA turnover rate and transport efficiency.

## Experimental procedures

### Molecular biology and cell culture

Human NKA α_1_, β_1_, and PLM were kindly provided by Prof. Steven J. D. Karlish (Weizmann Institute of Science). α_1_ subunit encoding cDNA was subcloned into the mCyRFP1-C1modified plasmid (Addgene) yielding α_1_ fused to the C-terminus of the mCyRFP1 (28), whereas PLM was subcloned into N1-mMaroon1 vector yielding PLM fused to N-terminus of mMaroon1 (29). The vectors containing mCyRFP1 and pcDNA3.1-mMaroon1 were purchased from Addgene. To improve NKA expression and localization, we coexpressed human β_1_ subunit in unlabeled-C1 vector with labeled α_1_ subunit in 1:1 ratio. All sequences have been verified by single pass primer extension analysis (ACGT Inc).

HEK293-AAV cells were incubated in the humidified 5% CO_2_ incubator in Dulbecco’s modified Eagle’s medium (DMEM), supplemented with 10% fetal bovine serum until 60 to 80% confluence. Cells were transiently transfected using Lipofectamine 3000 transfection kit (Invitrogen) and transferred into DMEM, supplemented with 10% fetal bovine serum and 2% dimethyl sulfoxide. Forty-eight hours posttransfection, cells were trypsinized, plated into sterile glass bottom chamber slides, and incubated at least 1 h before imaging. The media in the chamber slides was replaced by phosphate buffered saline immediately before imaging.

### Human heart tissue procurement

Human left-ventricular tissue was provided by Loyola Cardiovascular Research Institute Biorepository. The sample collection was approved by Loyola University Review Board (IRB number 210940821918), and written informed consent was obtained for the collection of heart tissue according to the Declaration of Helsinki. Tissue from failing human hearts with nonischemic idiopathic dilated cardiomyopathy was collected at the time of heart explant or at the time of LVAD implantation and flash frozen in liquid nitrogen.

### Human tissue membrane protein enrichment

Frozen left-ventricular tissue was placed in 5 ml of a buffer A (100 mM KCl, 2.5 mM K_2_HPO_4_, 2.5 mM KH_2_PO_4_, 2 mM EDTA) containing protease and phosphatase inhibitors (1:100 ratio). The tissue homogenized with a mechanical homogenizer and rotated for 1 h at 4 °C. Samples were centrifuged at 6400*g* for 20 min at 4 °C. The supernatant containing soluble proteins from the previous step was centrifuged at 10,000*g* for 20 min to remove heavy debris. Subsequently, the supernatant was centrifuged at 48,000*g* at 4 °C. Finally, the pellet containing the membrane proteins was dissolved in 100 μl of buffer B (1 M sucrose, 50 mM KCl) and stored at −80 °C. The total protein concentration was determined using Bicinchoninic Acid Kit for Protein Determination (Pierce), and all reagents were purchased from Sigma Aldrich if not stated otherwise.

### HEK cell membrane protein enrichment

Forty-eight hours posttransfection, cells were scraped in 5 ml homogenizing buffer composed of 250 mM sucrose, 10 mM Tris, 2 mM EDTA pH 7.4 with protease inhibitor and centrifuged cells for 10 min at 4000*g*. Cell pellet was resuspended in homogenizing buffer with protease inhibitors and homogenized. Lysed cells were centrifuged for 20 min at 4000*g*. Supernatant was collected and centrifuged at 55,000*g* for 30 min to collect total membrane fraction.

### Western blotting

After isolating the membrane fraction, protein concentration was determined using a bicinchoninic acid assay (Thermo Fisher). Samples were denatured in 4× Laemmli sample buffer with beta-mercaptoethanol at 90 °C for 5 min, run on a 4 to 15% polyacrylamide gradient gel (Bio-Rad) and transferred to polyvinylidene difluoride membrane. Following transfer, membranes were incubated with Revert Total Protein Stain (LI-COR Biosciences) for 5 min to obtain total protein in each lane and then blocked in a 1:1 ratio of Intercept Blocking Buffer (LI-COR Biosciences) to PBS with Tween solution for 1 h at room temperature. Afterward, membranes were incubated with primary antibody, anti-pan NKA from Development Studies Hybridoma Bank, University of Iowa (1:1000, cat. no. AB2166869) overnight in PBS with Tween at 4 °C with gentle rocking. Blots were incubated with anti-mouse secondary antibody for 1 h at room temperature (1:10,000 dilution) and analyzed using the LI-COR Image Studio software.

### Progressive acceptor photobleaching

Cells were transfected in 1:1, 1:3, and 1:5 ratios between donor-labeled α subunit and acceptor-labeled α, β, and PLM. Forty-eight hours posttransfection, cells were trypsinized and plated at 200,000 cells/well density into 2-well glass bottom chamber slides and incubated for 1 h at 37 °C. Immediately before imaging, cells were washed with PBS. Images were acquired with a Nikon Eclipse Ti2 inverted microscope with a 1.49 Apo TIRF 100× oil immersion objective and Nikon PFS system. A sequence of CFP and YFP images of the field of cells was imaged every 10 s before YFP photobleaching was applied. Acceptor was selectively photobleached using 30 s YFP exposure time and YFP, CFP sequence was recorded after each photobleaching step from a total of 50 bleaching steps.

### TCSPC and fluorescence lifetime imaging microscopy

TCSPC data acquisition was performed as previously described (23, 34). TCSPC histograms were obtained using HEK293-AAV cells, expressing mCyRFP1-α_1_ NKA alone or coexpressing mCyRFP-α_1_ NKA with PLM-mMaroon1 in 1:5 donor:acceptor ratio, which was described in the experimental protocol. Experiment using unlabeled PLM as a competitor used 1:4 ratio between donor and unlabeled acceptor and 1:1 ratio between donor and acceptor. Fluorescently labeled proteins were manually selected using 500 mm focal length plan-convex lens in a flip mount to defocus the excitation supercontinuum laser beam (Fianium) to excite the whole cell. To excite mCyRFP1, the excitation laser beam was filtered through 482/18 nm bandpass filter and 0.3 neutral density filter, and emitted fluorescence was detected using 640/50 nm bandpass filter. After selection of a cell for spectroscopy, the defocusing lens was removed from the light path, the laser intensity was attenuated with a 1.0 neutral density filter, and the laser focus was positioned on a region of the cell that yielded a fluorescence intensity of 100,000 photons/s. Under these excitation conditions, we observed less than 5% photobleaching during the acquisition period. Fluorescence was detected through a 1.2 N.A. water-immersion objective with avalanche photodiode and photon counting module (PicoHarp300, PicoQuant) using a time channel width of 16 ps. Sixty seconds of acquisition was performed for each cell, excluding cells that showed significant fluorescence intensity changes due to movement. Protein expression did not correlate well to photon count rate, which was determined primarily by how well the focused beam superimposed the plasma membrane, rather than the density of proteins in the bilayer. In several previous studies (22, 23, 34, 57), we circumvented this issue by defocusing the excitation laser to illuminate a larger portion of the microscopic field so that we could measure the fluorescence intensity of the whole cell with a camera as an index of protein expression level (23). This was less effective in the present study, as illuminating NKA required positioning of the focused beam on the plasma membrane, thus the rest of the cell was inconsistently positioned in the defocused area of illumination, compromising intensity measurements. Under these conditions, the donor-only decay was single-exponential (χ^2^ ranging from 0.961 to 1.059 for mCyRFP1-α_1_). The mCyRFP1-α_1_ construct showed a minor second component, but the amplitude of this component was only 5% so we considered it justifiable to approximate with a single exponential decay. In cells expressing donor- and acceptor-labeled proteins, additional shorter lifetimes were observed, which we attributed to FRET. Fluorescence decay histograms were analyzed using global analysis in SymPhoTime 64 software with fixed lifetime for donor alone (3.46 ns) and two freely variable global lifetimes and all variable amplitudes for all lifetimes. Analysis of the distribution of acceptors in NKA–PLM complexes in Figure 2*E* was performed using global analysis with shared lifetime and freely variable amplitude for FRET species. We observed no differences for α subunit isoforms, so the data were combined and analyzed collectively. Distances corresponding to obtained lifetimes were calculated assuming an orientation factor (κ^2^) of 2/3 and using the relationship $R=R_{0}\sqrt[{6}]{\left(\frac{1}{E}-1\right)}$ (58), where R_0_ is the Förster distance for mCyRFP1-mMaroon1 pair (63.34 Å) (29) and E is average FRET efficiency from lifetime measurements.

Relative protein concentrations were interpolated from maximal FRET efficiency determined using TCSPC with mCyRFP1 and mMaroon1 and FRET_max_ obtained from hyperbolic fit of acceptor sensitization FRET-binding curve of constructs labeled with CFP and YFP. The mMaroon1 protein concentration was calculated using following relationship $\left[mMaroon1\right]=\frac{K_{D\;}AverageFRET}{\left(FRET_{max}-AverageFRET\right)}$, where Average FRET is equal to 1 − (τ_DA_/τ_D_), τ_DA_ is lifetime of donor in the presence of acceptor, τ_D_ is lifetime of donor alone, FRET_max_ is the value of maximal FRET efficiency detected for all cells in the dataset (74.47% in our case) and K_D_ is dissociation constant.

### Sample size estimation and number of replicates

Data were obtained from at least four independent transfections without prior estimation of the sample size. TCSPC analysis was performed on sample size of 10 to 80 cells. Fluorescence lifetime imaging microscopy analysis of isoform specific interactions was performed on sample size of 24 to 64 cells. Acceptor sensitization FRET experiments used 500 to 900 cells per replicate, and 4 to 16 independent replicates were analyzed. Progressive acceptor photobleaching experiments used 15 to 40 individual cells per replicate, where at least 4 replicates were analyzed.

### Statistics

Data are represented as mean ± standard error of the mean of n measurements. Statistical comparison between two groups was performed in OriginPro2020b software (OriginLab) using Student’s *t* test for unpaired data sets. The differences considered as statistically significant had *p* < 0.05.

### Acceptor sensitization measurements

This experiment was performed as previously described in (34, 59) with some modifications. Similar to progressive acceptor photobleaching experiment, cells were transiently transfected in 1:5 ratio between donor and acceptor, trypsinized 48 h posttransfection and plated at 100,000 cells/well density into 4-well glass bottom chamber slides, and incubated for 1 h at 37 °C. Subsequently, cells were washed twice with PBS. Images of cells were acquired by Nikon Eclipse Ti2 inverted microscope with NA 40× air objective. Acceptor-sensitized FRET was used to measure the binding affinity of mCer3-NKA and PLM-eYFP constructs. For each field, the set of four images of CFP, YFP, CFP-YFP FRET/fluorescence were acquired using following exposure times of 500 ms, 100 ms, and 500 ms, respectively. Specific fluorophore was excited with adequate excitation wavelength with subsequent emission detection for CFP and YFP channels with the exception of CFP-YFP FRET where excitation wavelength for CFP was used and YFP emission was captured. Then, stage was shifted to a new position and another field was imaged without overlapping areas. To ensure that cells are remaining in focus, Nikon PFS feedback system was used. The data were analyzed using automated cell scoring algorithm in Metamorph software using YFP channel for identification of cells for scoring. Cells with signal higher than background in all three (CFP, YFP, and FRET) channels were analyzed.

### Molecular docking of NKA dimers

To explore the atomic details of the NKA dimer interface, we performed molecular docking of NKA dimers in different states of the reaction cycle using the following crystal structures: 3KDP (E2P) (60), 7DDJ (E2Pi) (61), and 3WGU (E1Pi.ADP) (40). Docking was performed following the strategy described in (41), in search of interactions between either homodimers or heterodimers (with 3KDP (60) in the E2P state). Specifically, we used ClusPro for global docking to generate possible starting structures. Following that, we used Rosetta MPDock for local refinement of these structures.

The ClusPro web server (62, 63, 64, 65) was first used to perform global docking of NKA dimers using default settings. The starting structures were stripped of hetero atoms and only the protein chains were used. Around 100 resulting structures were then screened based on the following: (1) whether the monomers are in parallel alignment (*i.e.*, the angle between the monomers should not exceed 40°) and (2) whether the transmembrane domains of each monomer were located within the same plane (*i.e.*, the distance between the transmembrane regions of the monomers should not exceed 5 Å). Dimer pairs that met the above criteria were next subjected to local refinement using Rosetta MPDock, a docking algorithm that runs protein-protein docking in the membrane bilayer (42, 66, 67). The general Rosetta MPDock protocol consists of three steps: (1) generate a membrane span file that defines the transmembrane regions of the proteins; (2) prepack the docking partners to optimize side chain orientation; and (3) protein-protein docking in the membrane environment. As a constantly updated database of transmembrane proteins from PDB, PDBTM provides not only protein coordinates but also transmembrane profiles of the proteins (68, 69, 70). The span files for the crystal structures were therefore generated based on PDBTM coordinates files except for 7DDJ, for which such files were not available. Accordingly, the transmembrane region of 7DDJ was defined by using the transmembrane domain from 3KDP as they share similar structures in that domain (RMSD = 1.506 Å). For each dimer pair, Rosetta MPDock generated 1000 structures. These 1000 structures were ranked by the Rosetta interaction energy (I_sc). We compared best ten conformations with our experimental data and selected three candidates with best agreement between docked structures and experimental measurements. All those three candidates represented 3WGU-3WGU combination and were used as initial models for MD simulations.

### MD simulations

We modeled NKA residues Glu327, Glu779, Asp804, and Glu954 as protonated (71), and we further kept two 1,2-diacyl-sn-glycero-3-phosphocholine molecules and cholesterol embedded between α and β subunits as in 3WGU structure to preserve the structural stability of the E1 state. The complexes were inserted in a pre-equilibrated 150 × 150 Å bilayer of POPC:POPE:POPS lipids (2:1:1) to mimic the composition of the plasma membrane. In this study, we used the replacement method to generate lipid packing around the αβ-FXYD2 dimers. The initial systems were solvated using TIP3P water molecules with a minimum margin of 25 Å in the z-axis between the edges of the periodic box and the protein. Na^+^, K^+^, and Cl^−^ ions were added to neutralize the system and to produce Na^+^ and K^+^ concentrations of 140 and 5 mM, respectively (72, 73). Molecular simulations were performed with AMBER20 on Tesla V100 GPUs (74) using the AMBER ff19SB force field (75). We maintained a temperature of 310 K with a Langevin thermostat and a pressure of 1.0 bar with the Monte Carlo barostat. We used the SHAKE algorithm to constrain all bonds involving hydrogens and allow a time step of 2 fs. The systems were subjected to 5000 steps of steepest-descent energy minimization followed by equilibration as follows: two 25-ps MD simulations using a canonical ensemble (NVT), one 25-ps MD simulation using an isothermal–isobaric ensemble (NPT), and two 5-ns MD simulations using the NPT ensemble. The equilibrated were used as a starting point to perform a single MD trajectory of each complex for a total simulation time of 2.6 μs using an NPT ensemble.

## Data and software availability

All code written in support of this publication are publicly available at https://github.com/huskeypm/pkh-lab-analyses. Docking input files and generated data are available upon request.

## Supporting information

This article contains supporting information.

## Conflict of interest

The authors declare that they have no conflicts of interest with the contents of this article.
