# Supplementary material for: Fluorescence lifetime imaging microscopy reveals sodium pump dimers in live cells
Source: J Biol Chem. 2022 Mar 24;298(5):101865. doi: 10.1016/j.jbc.2022.101865 (PMC9048134; doi:10.1016/j.jbc.2022.101865)
Supplement: Supplemental Figures S1–S6 [file mmc1.pdf]

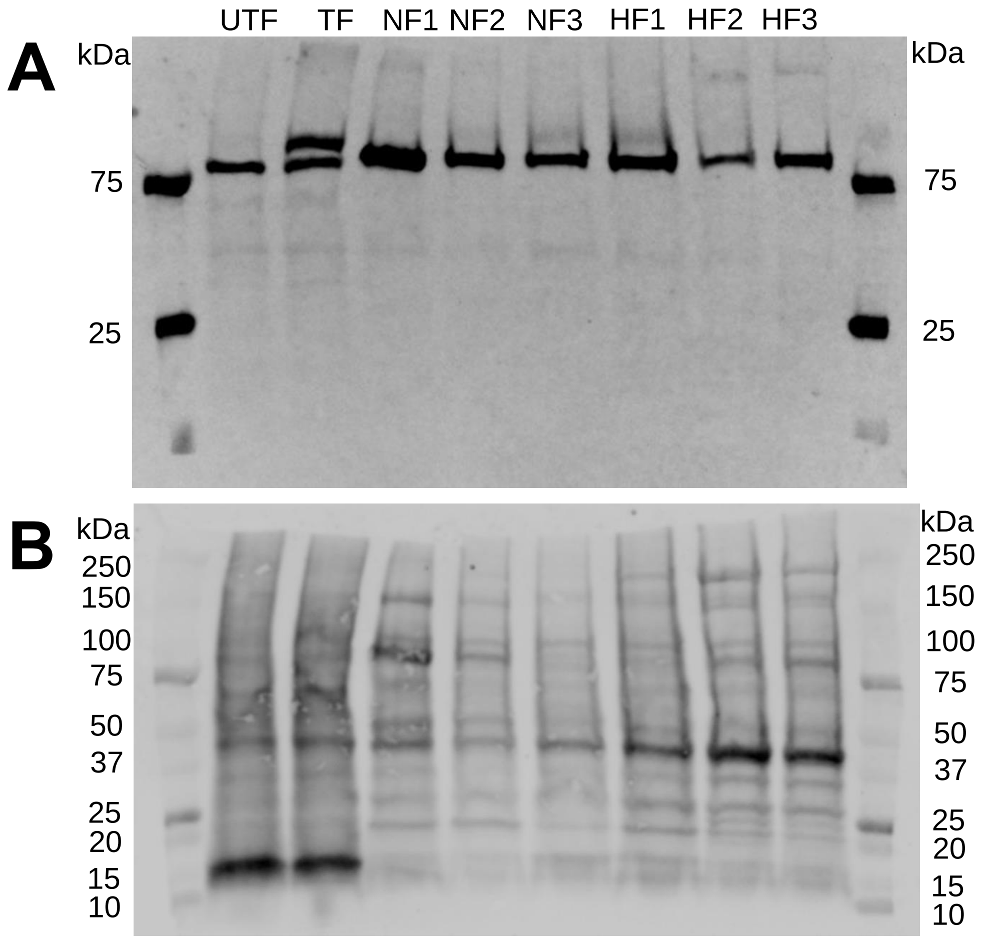

Sup. Fig. 1: Uncropped Western blot (as in Fig. 1) for analysis of sodium pump expression in heterologous cells and myocardium. **(A)** Un-transfected HEK cell microsomal fractions (UTF) showed endogenous NKA with an apparent MW of 100 kDa. Microsomes from cells transfected with human mCer-α_1 NKA (TF) showed bands representing endogenous NKA and exogenous fluorescent NKA, which showed a decreased mobility due to the 30 kDa tag. NKA was highly expressed in microsomal fractions isolated from human myocardium. We observed decreased expression of NKA in failing human heart (HF1-3) compared to non-failing hearts (NF1-3). **(B)** Total protein staining (Revert). TF and UTF data represent 3 independent transfections. NF and HF data represent tissue samples from 8 non-failing donor hearts and 8 explanted hearts with dilated cardiomyopathy.

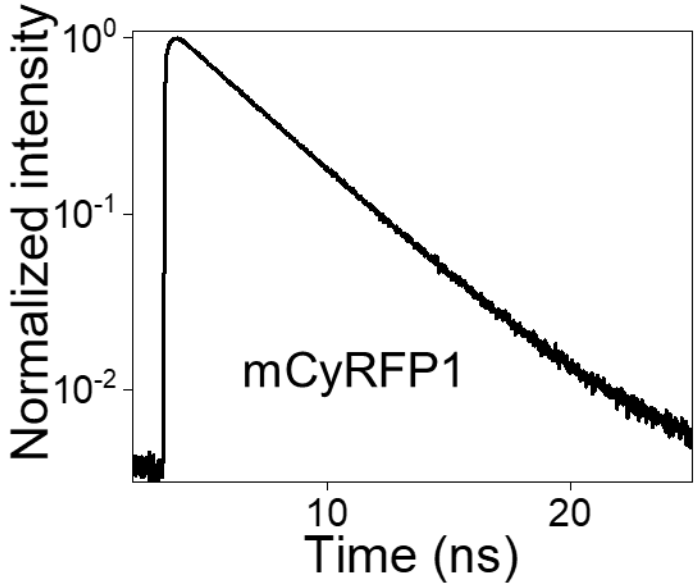

Sup. Fig. 2: Quantification fluorescence lifetime by TCSPC. Free (non-fusion) mCyRFP1 protein decays with single exponential kinetics.

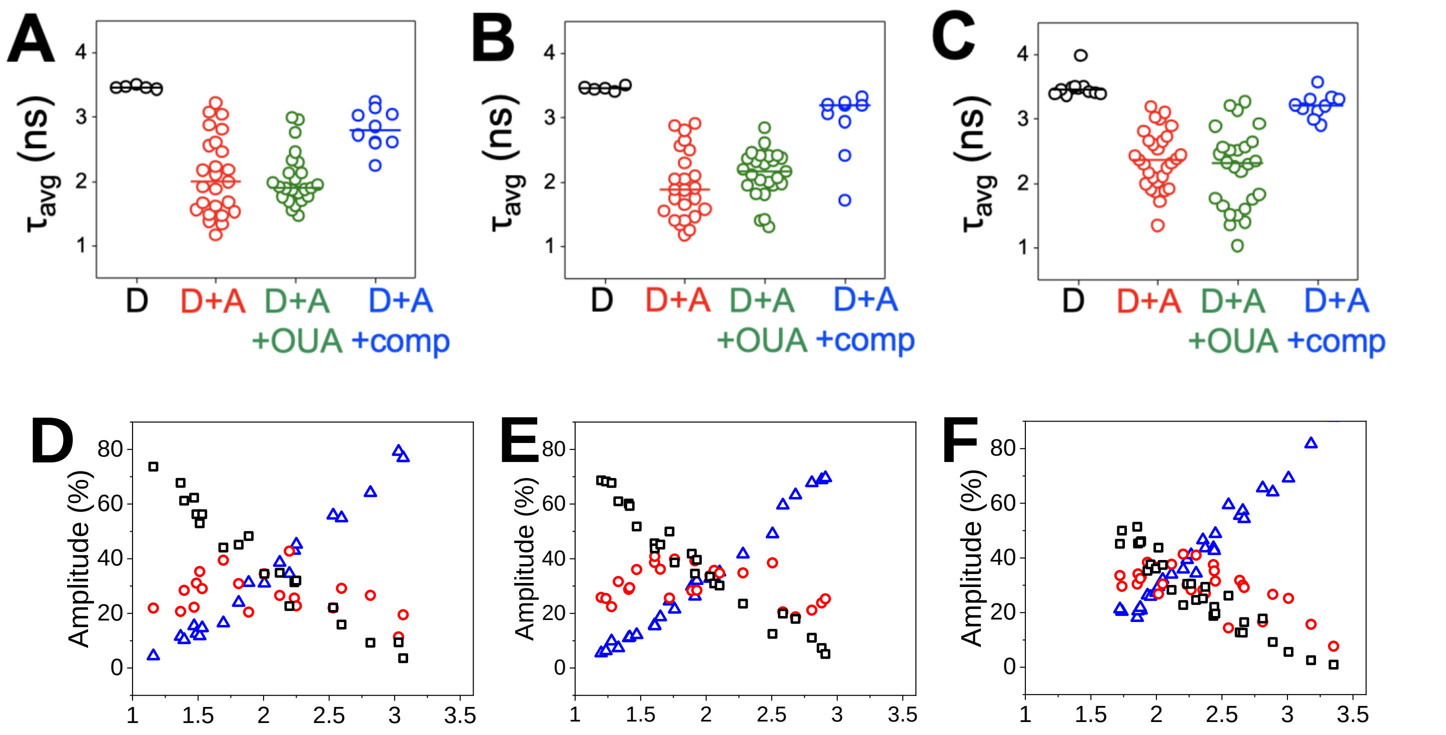

Sup. Fig. 3: Quantification of fluorescence decays. (**A**) A decreased fluorescence lifetime indicates FRET between donor-labeled α_1 (D) and acceptor labeled PLM (A). FRET was not affected by addition of ouabain (OUA). FRET was reduced by co-expression of unlabeled PLM. (**B**) As in (A), but with the α_2 isoform. (**C**) As in (A), α_3 isoform. (**D**) The relative population of FRET species for α_1. (**E**) The relative population of FRET species for α_2. (**F**) The relative population of FRET species for α_3. We observed no differences for α subunit isoforms, so the data were combined and analyzed collectively.

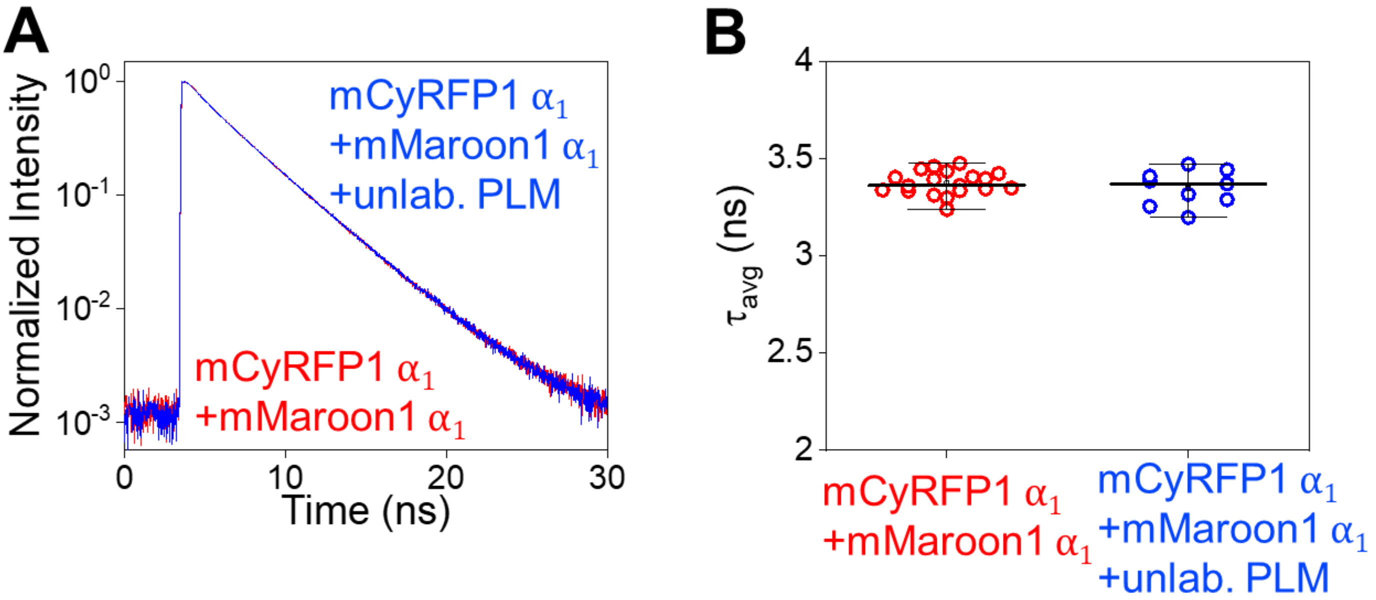

Sup. Fig. 4: Quantification of changes in NKA-PLM regulatory complex caused by the presence of unlabeled PLM detected by TCSPC. (**A**) The fluorescence decay of α-α pair (red) and α-α-unlabeled PLM (blue). Data are averages of 10 (for α-α) or 9 (for α-α+PLM) decay curves (**B**) Average fluorescence lifetime for mCyRFP1-α_1 co-expressed with mMaroon1-α_1. (red circles) and the same pair of the donor and acceptor in the presence of unlabeled PLM as a competitor (blue circles). Presence of the unlabeled PLM does not affect α-α FRET.

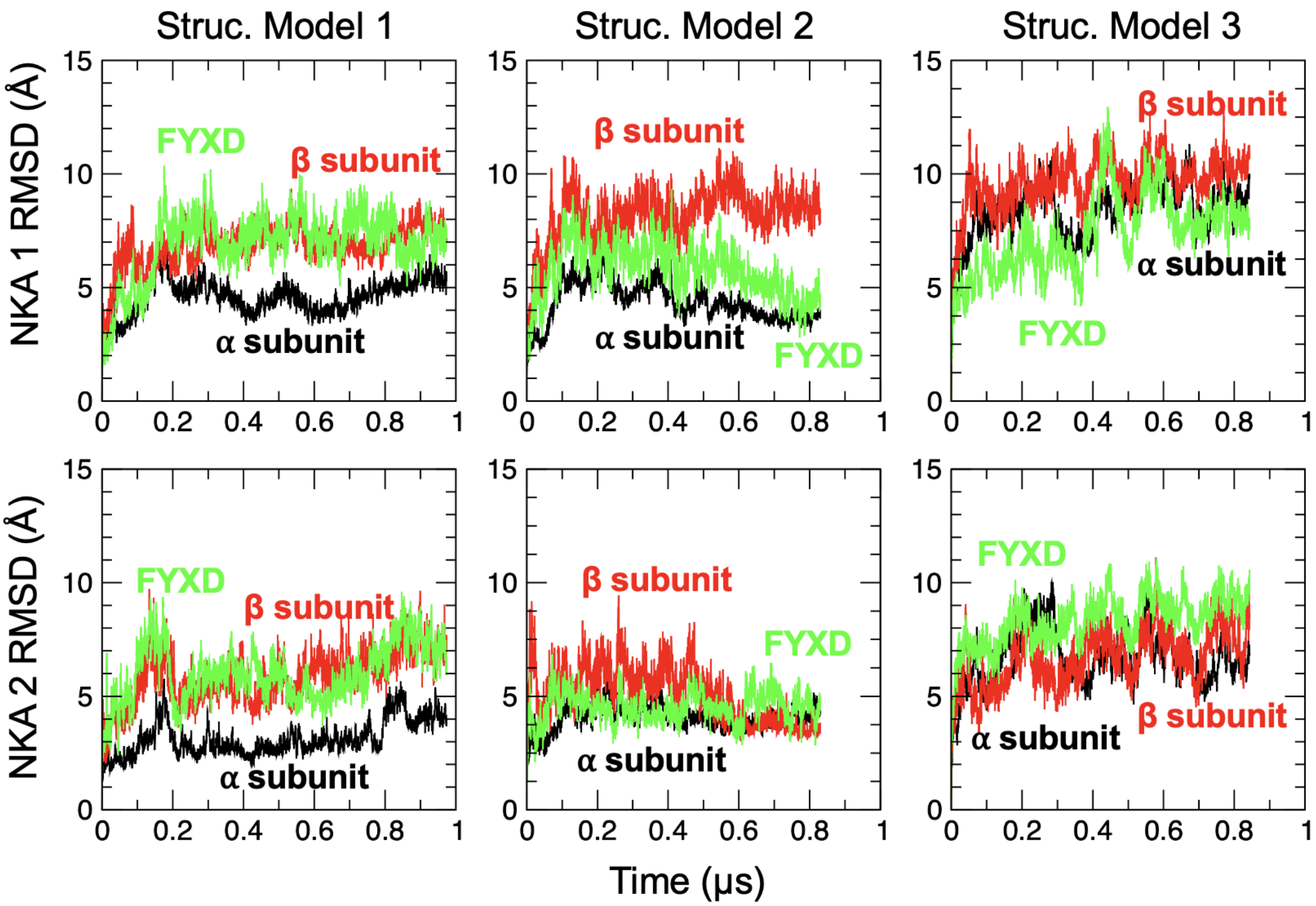

Sup. Fig. 5: RMSD for α (black), β (red), and FYXD (green) was calculated by aligning the backbone of the entire dimer with the structure at the beginning of each MD trajectory.

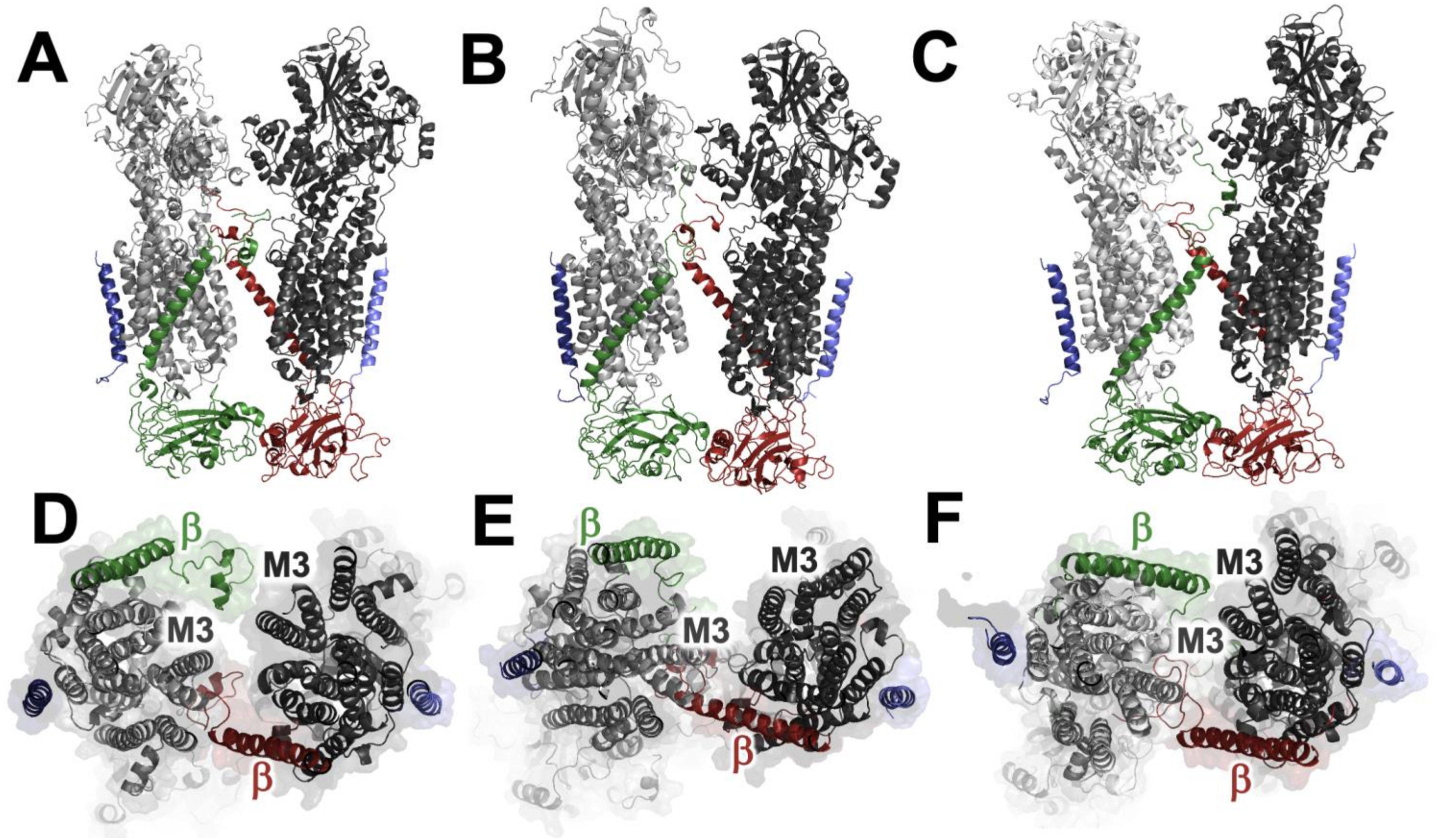

Sup. Fig. 6: Structures of alternative Structural Models 1, 2, and 3, showing α subunits in gray, β subunits in red and green, and FXYD proteins in blue or lavender.
